# Correlating Infant Fecal Microbiota Composition and Human Milk Oligosaccharide Consumption by Microbiota of 1‐Month‐Old Breastfed Infants

**DOI:** 10.1002/mnfr.201801214

**Published:** 2019-04-30

**Authors:** Klaudyna Borewicz, Fangjie Gu, Edoardo Saccenti, Ilja C.W. Arts, John Penders, Carel Thijs, Sander S. van Leeuwen, Cordula Lindner, Arjen Nauta, Ellen van Leusen, Henk A. Schols, Hauke Smidt

**Affiliations:** ^1^ Laboratory of Microbiology Wageningen University & Research Stippeneng 4 6708 WE Wageningen The Netherlands; ^2^ Laboratory of Food Chemistry Wageningen University & Research Bornse Weilanden 9 6708 WG Wageningen The Netherlands; ^3^ Laboratory of Systems and Synthetic Biology Wageningen University & Research Stippeneng 4 6708 WE Wageningen The Netherlands; ^4^ Department of Epidemiology CAPHRI Care and Public Health Research Institute Maastricht University Minderbroedersberg 4–6 6211 LK Maastricht The Netherlands; ^5^ Department of Epidemiology CARIM School for Cardiovascular Diseases, Maastricht University Universiteitssingel 50 6229 ER Maastricht The Netherlands; ^6^ Maastricht Center for Systems Biology (MaCSBio) Universiteitssingel 60 6229 ER Maastricht The Netherlands; ^7^ Department of Medical Microbiology Maastricht University Medical Centre P. Debyelaan 25 6229 HX Maastricht The Netherlands; ^8^ NUTRIM School for Nutrition, Toxicology and Metabolism Universiteitssingel 40 6229 ER Maastricht The Netherlands; ^9^ Microbial Physiology, Groningen Biomolecular Sciences and Biotechnology Institute (GBB) University of Groningen Nijenborgh 7 9747 AG Groningen The Netherlands; ^10^ FrieslandCampina Innovation Centre Bronland 20 6708 WH Wageningen The Netherlands

**Keywords:** breastfeeding, human milk oligosaccharide, microbial clusters, microbiome

## Abstract

**Scope:**

Understanding the biological functions of human milk oligosaccharides (HMOs) in shaping gastrointestinal (GI) tract microbiota during infancy is of great interest. A link between HMOs in maternal milk and infant fecal microbiota composition is examined and the role of microbiota in degrading HMOs within the GI tract of healthy, breastfed, 1‐month‐old infants is investigated.

**Methods and results:**

Maternal breast milk and infant feces are from the KOALA Birth Cohort. HMOs are quantified in milk and infant fecal samples using liquid chromatography‐mass spectrometry. Fecal microbiota composition is characterized using Illumina HiSeq 16S rRNA gene amplicon sequencing. The composition is associated with gender, delivery mode, and milk HMOs: Lacto‐*N*‐fucopentaose I and 2′‐fucosyllactose. Overall, *Bifidobacterium, Bacteroides, Escherichia–Shigella*, and *Parabacteroides* are predominating genera. Three different patterns in infant fecal microbiota structure are detected. GI degradation of HMOs is strongly associated with fecal microbiota composition, and there is a link between utilization of specific HMOs and relative abundance of various phylotypes (operational taxonomic units).

**Conclusions:**

HMOs in maternal milk are among the important factors shaping GI tract microbiota in 1‐month‐old breastfed infants. An infant's ability to metabolize different HMOs strongly correlates with fecal microbiota composition and specifically with phylotypes within genera *Bifidobacterium, Bacteroides*, and *Lactobacillus*.

## 1 Introduction

During and after birth, microorganisms from the mother and other environmental sources colonize an infant. Genetic and various environmental factors and life events further shape the microbial communities, making them specific to each body site and to each individual. These microbial ecosystems acquired and developed in early life play an important role in well‐being and health, both during infancy and beyond.^1^ One of the body sites that undergoes a rapid microbial colonization in early life is the gastrointestinal (GI) tract.^1^ The anaerobic conditions in the lower GI tract favor the establishment of bacteria, such as *Bifidobacterium*, *Bacteroides*, and *Clostridium*.^1^ Besides the absence of oxygen, diet is another key factor that has a strong influence on shaping the GI microbial ecosystem.

In breastfed infants, breast milk is the sole source of nourishment during the first few months of life. Breast milk is a complex biofluid that contains high concentrations of lactose, lipids, proteins, and milk glycans, the latter being present as either glycoproteins or free human milk oligosaccharides (HMOs).^2^ HMOs play an important role in intestinal cell proliferation and maturation, maintaining epithelial barrier function, and protecting the GI tract against bacterial and viral pathogens and toxins.^2^, ^3^, ^4^, ^5^ Despite being the third most abundant component of human milk, HMOs are not affected by infant digestive enzymes.^2^ As a result, milk HMOs reach the infant colon, where they are degraded by bacteria. Since not all bacteria have the necessary enzymes to utilize HMOs, these milk glycans facilitate the establishment of a highly specialized microbial ecosystem dominated by bifidobacteria and *Bacteroides* among others, while indirectly limiting growth of other bacteria. This prebiotic effect has been demonstrated for selected bacterial species, both in vitro^3^, ^6^ and in vivo,^7^ and it has been recognized as one of the key drivers for bacterial species succession in the infant GI tract.^5^

Maternal genotype (e.g., mother's secretor status) determines the HMO composition in breast milk, and the concentrations of different HMOs vary between individuals and across lactation stages.^8^, ^9^, ^10^ This variability might target the distinct and changing needs of a growing infant and orchestrate the stepwise development of infant GI tract microbiota. Recent developments in glycomics led to the recognition of over 200 different HMOs in human milk.^2^ The core structures of HMOs include galactose, glucose, and *N*‐acetyl‐glucosamine, which are further decorated with fucose and/or sialic acid. The fucosylation of HMOs depends on the presence of specific glycosyltransferases, including the α1‐2‐fucosyltransferase FUT2 and the α1‐3/4‐fucosyltransferase FUT3, in lactating women. Milk of secretor women having active *Se* gene locus that encodes the FUT2 has high amounts of α1‐2‐fucosylated HMOs, while milk of Lewis‐positive women with active *Le* gene locus that encodes the FUT3 is abundant in α1‐4‐fucosylated HMO. In contrast, milk of non‐secretor or Lewis‐negative women lack α1‐2‐ or α1‐4‐fucosylated HMO, respectively.^11^ Based on the presence or absence of sialic acid, HMOs can be classified into two categories: the neutral and the acidic HMOs. In this study, we measured 17 highly abundant HMOs, including 12 neutral and 5 acidic HMOs (Table S1, Supporting Information). The concentrations of these major HMO structures account for around 86% of the total HMOs in breast milk of secretors, 35 days postpartum.^12^

In the light of growing evidence supporting the role of the early colonization of GI tract microbial ecosystem in health, understanding the biological function of the different HMOs is of great interest. Previous studies focused mainly on in vitro fermentation of HMOs by fecal bacterial inoculum, or by fecal isolates.^3^ A proof‐of concept study showed correlation between HMO degradation and gut microbiota development in early life by analyzing samples from two infants.^12^ However, the HMO degradation within an infant GI tract is still not fully understood. Here, we analyzed 121 mother–infant pairs to investigate the association between selected maternal HMOs and the infant fecal microbiota composition. Our two main research questions were i) whether there was an association between the composition of HMOs in breast milk and the composition of fecal microbiota in healthy, breastfed, 1‐month‐old infants, and ii) if the degradation of these HMOs could be linked to infant fecal microbial communities and to specific bacterial taxa found in the infant's GI tract.

## 2 Experimental Section

### 2.1 Milk and Fecal Sample Collection

The milk and fecal samples used in this study originated from the KOALA Birth Cohort (Dutch acronym for Child, Parents and Health: Lifestyle and Genetic Constitution). The design, selection criteria, and feces collection procedure have been described elsewhere, and the study was approved by the Ethics Committee of the University Hospital of Maastricht.^13^, ^14^, ^15^ Briefly, the KOALA study included healthy pregnant women living in the south of the Netherlands (*N* = 2834), and the exclusion criteria included prematurity (birth before 37 weeks of gestation), twins, congenital abnormalities related to growth, and administration of antimicrobial agents before feces collection. Only infants who were exclusively breastfed, and for whom both the fecal and the corresponding maternal breast milk samples were available were included in the analyses (*n* = 121). All infants were born in the years 2002–2003, healthy, full term, at home or hospital via either vaginal delivery or C‐section. Three infants were reported by the parents as sick on the sample collection day, but none of the infants received antibiotics during the first month of life (Tables S1 and S2, Supporting Information). Infant fecal samples were collected at approximately 1 month postpartum from infants’ diapers, refrigerated, and sent to the lab by post within 1 day after collection. Fecal samples were stored in peptone glycerol solution (10 g L^−1^ peptone water in 20 v/v% glycerol) at 1 g of feces in 9 mL of solution. Breast milk samples were collected into sterile tubes (Cellstar PP‐test tubes, Kremsmünster, Austria) in the morning on the same day as the fecal samples, refrigerated (±4 °C), transported on ice, and processed in the lab on the same day. Breast milk samples were centrifuged (400 × *g*, 12 min, no brake, 4 °C) and the lipid and aqueous fraction were separated and stored in plastic vials (Sarstedt, Nümbrecht, Germany) at −80 °C in the European Biobank, Maastricht. The remaining debris was not used to avoid contamination with cell fragments.

### 2.2 Dosage Information/Dosage Regime

The total amount of HMOs ingested daily by each infant depended on the HMO concentration in breast milk and the total amount of breast milk consumed per day. The daily intake was estimated based on the literature data on infants of similar age (approximately 1 month postpartum) who were breastfed without feeding problems and consumed amounts as regulated according to their needs.^16^

### 2.3 DNA Extraction , PCR and sequencing

Total DNA was extracted from the stool samples, as previously described,^17^ using the double bead‐beating procedure followed by the QIAamp DNA stool mini kit (Qiagen, Hilden, Germany) according to the manufacturer's instructions. The resulting DNA templates (5–20 ng) were used for subsequent PCR amplification and Illumina HiSeq sequencing of the V4 region of 16S ribosomal RNA (rRNA) genes.^18^

### 2.4 HMO Analysis

HMOs were isolated and purified from milk and infant feces using solid phase extraction.^19^ Then, HMOs were reduced to alditols using sodium borohydride^20^, ^21^ and were analyzed by an Accela Ultra High Pressure Liquid Chromatography (UHPLC) system (Thermo Scientific, Waltham, MA, USA), which was coupled to a Velos Pro mass spectrometer (Thermo Scientific) with an electrospray ionization probe. A volume of 5 µL of reduced sample or HMO commercial standard was injected onto a Thermo Hypercarb Porous Graphitic Carbon LC Columns column (100 × 2.1 mm, 3 µm particle size) with a Hypercarb guard column (10 × 2.1 mm, 3 µm particle size). The separation was performed using two eluents: A) 1% v/v ACN in water with 0.1% v/v formic acid and B) ACN with 0.1% v/v formic acid. The elution profile was applied as follows: 0–5 min, 3% B; 5–22 min, 3–20% B; 22–32 min, 20–40% B; 32–33 min, 40–100% B; 33–43 min, 100% B; 43–44 min, 100–3% B; 44–65 min, 3% B. In total, 12 neutral HMOs (2′FL, LNT and LNnT, 3FL, DFL, LNDFHI, LNFPI, LNFPII, LNFPIII, LNFPV, LNH, LNnH) and 5 acidic HMOs (3′SL, 6′SL, LSTa, LSTb, LSTc) were measured. Data processing was done by using XCalibur software (Thermo Scientific), and peak area as extracted from MS signal was used for quantitation. Quantitation of 3FL was by high‐performance anion exchange chromatography–pulsed amperometric detection (HPAEC‐PAD). The analysis was applied on an ICS 5000 system (Dionex, Sunnyvale, CA), equipped with a CarboPac PA‐1 column (250 mm × 2 mm ID) and a CarboPac PA guard column (25 mm × 2 mm ID) and with a column temperature of 20 °C. The two mobile phases were A) 0.1 m NaOH and B) 1 m NaOAc in 0.1 m NaOH. With a flow rate of 0.3 mL min^−1^, the gradient elution profile was as follows: 0–10 min, 0–10% B; 10–10.1 min, 10–100% B; 10.1–15 min, 100% B; 15–15.1 min, 100–0% B; 15.1–30 min, 0% B. The elution was monitored by a pulsed amperometric detector (Dionex ICS‐5000 ED). Data processing from HPAEC was done by using Chromeleon 7.1 software (Dionex). The HMOs concentrations were measured in micrograms per milliliters of milk or micrograms per gram of feces.

### 2.5 Data Analysis

The 16S rRNA gene sequencing data were analyzed using the NG‐Tax analysis pipeline using default settings.^22^ In brief, libraries were filtered to contain only read pairs with perfectly matching barcodes that were subsequently used to separate reads by sample. Operational taxonomic units (OTUs) were assigned using an open reference approach and SILVA_111_SSU 16S rRNA gene reference database (https://www.arb-silva.de/).^23^ Microbial composition data were expressed as a relative abundance of each OTU obtained with NG‐Tax.

Infants were classified into three distinct microbial cluster types based on genus level microbial abundance data using Dirichlet Multinomial Mixture (DMM) modeling.^24^ Briefly, the number of Dirichlet components was selected by inspection of the fit of the model to the count data for varying number of components (1 to 7). Goodness of fit was assessed using the Laplace and the Akaike information criteria. Finally, each sample was assigned to the component for which it had the largest fitted value using the DirichletMultinomial R package^25^ in R (version 3.3.1). Microbial composition of each DMM cluster is shown in Figure S1, Supporting Information.

Redundancy analysis (RDA) was done in Canoco5^26^ using the log transformed OTU level relative abundance data with significance assessed using a permutation test. Explanatory variables included concentrations of milk HMOs: 2′FL, LNT and LNnT, LNFPIII, LNFPII, LNFPI, LNFPV, LNH, LNnH, LNDFHI, DFL, 6′SL, 3′SL, LSTc, LSTb, LSTa, 3FL, delivery mode (normal vaginal, assisted vaginal, and C‐section), delivery place (home, hospital), gender, gestational age, mother's antibiotic use, infant's signs of sickness (more specifically, the signs of gastroenteritis including vomiting, fever, and diarrhea) at the time of sample collection, infant age in days, and birth weight. The association between fecal microbiota composition, the assignment of each infant to a specific microbial cluster, and the HMO concentrations in corresponding breast milk samples of the infant's mother were investigated with partial least squares regression (PLS) using MatlabR2107a, and resulting *p*‐values were corrected for multiple comparisons using FDR. The Chi‐square test was used to assess the significance of the association between infant's gender and infant's microbial cluster type, and between mother's secretor status (positive, negative) and infant's microbial cluster type.

HMO degradation (consumption) in the infant GI tract was estimated based on profiles in breast milk and corresponding infant feces. Based on the utilization of the 17 individual HMOs, infants were assigned to consumption categories: “Complete,” “Non‐specific,” and “Specific” (acidic, neutral, or other). The Chi‐square test was used to assess the significance of the association between the assigned consumption category for each HMO and the microbial DMM cluster type of each infant.

Based on the extent to which each individual HMO was consumed (calculated as a ratio of the HMO concentration in infants’ feces and the concentration of the same HMO measured in mothers’ milk), infants were divided into tertiles (“low,” “medium,” or “high”) for consumption levels of each individual HMO. If a given HMO was not detected in milk, the consumption score was not included in the analysis, and if the amount in feces exceeded the amount detected in milk, the infant was assigned to the “low” category for that HMO. Associations between fecal microbiota composition and the assignment of each infant to a “low,” “medium,” or “high” consumption category for each HMO were investigated with RDA analysis in Canoco5, with significance assessed using a permutation test.^26^ Kruskal–Wallis analysis was performed in QIIME^27^, ^28^ to identify bacterial OTUs that differed significantly between infants who were classified as “high,” “medium,” and “low” consumers for each individual HMO.

### 2.6 Nucleotide Sequences

KOALA data sets cannot be made publicly available due to data confidentiality and the potential to identify individual study participants from the data. Data are available to the research community through the Dataverse repository (https://dataverse.nl/; 10411/CEGPGR) upon request to Prof. C. Thijs of the KOALA Study Management Committee at Department of Epidemiology, Maastricht University, PO Box 616, 6200 MD Maastricht, The Netherlands; e‐mail: c.thijs@maastrichtuniversity.nl, tel: +31(0)43 3882374.

## 3 Results

### 3.1 HMO Analyses

HMOs in maternal milk and in infant feces were quantified. The minimum, maximum, median, average, and standard deviation of the concentrations of each HMO, the HMO type (neutral, fucosylated, and sialylated), and the total amounts are summarized in **Table** 1. Total concentrations of the measured HMOs in milk ranged from 2.0 to 6.5 mg mL^−1^ and were lower than those reported in literature.^11^, ^29^, ^30^ We observed large individual variation in the HMO concentrations in both the maternal milk samples and in infant feces. In most samples, the percentage of neutral HMOs was higher than that of acidic HMOs (Table 1). The fucosylated HMOs accounted for the vast majority of neutral HMOs. The composition of fucosylated HMOs in milk depends on the Lewis and secretor status of a mother, and in some samples, we did not detect fucosylated HMOs at all. Structures like 2′FL and LNFP I were present in high concentrations in milk of secretors, while they were absent in non‐secretors. Other structures, like LNFP II, were present as main HMOs in the Lewis‐positive mothers, while they were absent in the Lewis‐negative mothers. We could not detect LNDFH I and DFL in breast milk samples containing α1‐2‐ and α1‐4‐fucoses from mothers who were Lewis or secretor negative. Some neutral HMOs were detected in all milk samples, that is, 3′FL, LNFP III, LNT, and LNnT. Only one mother lacked LNH and LNnH in her milk. The acidic HMOs were detected in all milk samples. These observations were close to those published before.^19^, ^29^ Similar to the HMOs in breast milk samples, there was also a large variation in HMO concentrations in the infant feces. However, the median values of milk HMO concentration matched well with the average values, while this was not the case for the HMO levels in feces, indicating a right‐skewed distribution for the fecal profiles that was different from the bell‐shaped distribution observed for the milk HMO profiles (Table 1).

**Table 1:** Average, minimum, maximum, and median concentrations of individual HMOs, classes, and total measured HMOs and corresponding standard deviations (SD), in breast milk and in infants’ feces solution (1 g feces per 9 mL of peptone glycerol medium)

	Concentrations of HMO or HMO category									
	Maternal breast milk [µg mL^−1^]					Infant fecal solution [µg mL^−1^]				
HMO	Min	Max	Median	Average	SD	Min	Max	Median	Average	SD
**3FL**	5.0	1098.0	182.0	248.0	222.0	NA	NA	NA	NA	NA
**2′FL**	0.0	852.8	460.0	372.7	242.3	0.0	240.3	0.5	29.6	61.4
**LNT and LNnT**	214.2	1806.7	948.0	976.2	319.0	0.0	372.7	15.8	48.6	75.0
**LNFPIII**	50.7	758.0	243.9	270.1	140.7	0.0	726.7	0.0	41.0	98.0
**LNFPII**	0.0	1341.5	236.3	339.0	294.3	0.0	549.0	3.1	81.9	125.5
**LNFPI**	0.0	1493.7	517.2	467.3	367.5	0.0	505.7	0.0	41.8	91.3
**LNFPV**	0.0	191.4	27.7	41.8	50.1	0.0	75.0	0.0	2.5	8.7
**LNH**	0.0	313.0	89.5	105.0	64.1	0.0	161.2	0.0	4.4	17.3
**LNnH**	0.0	299.1	56.1	72.2	56.3	0.0	563.4	0.4	12.1	57.0
**LNDFHI**	0.0	1856.2	548.3	475.5	388.3	0.0	889.8	28.0	204.3	258.2
**DFL**	0.0	125.9	42.1	40.4	32.1	0.0	68.4	0.6	9.7	17.0
**6′SL**	16.6	385.7	97.3	110.8	63.5	0.0	298.5	0.1	18.4	46.7
**3′SL**	16.8	194.8	91.5	90.7	38.8	0.0	100.0	0.0	4.2	14.9
**LSTc**	14.7	334.1	98.8	116.2	68.8	0.0	248.5	0.8	28.9	56.8
**LSTb**	53.2	804.4	244.4	256.2	118.7	0.0	499.9	1.8	52.2	104.5
**LSTa**	6.3	83.4	24.6	28.2	15.4	0.0	31.7	0.0	2.1	6.1
**Sum neutral**	1542.0	5717.3	3064.8	3160.2	824.2	0.0	1671.6	237.9	475.9	523.1
**Sum fucosylated**	266.5	4489.4	2042.5	2006.8	737.6	0.0	1591.7	186.1	410.8	463.4
**Sum sialylated**	174.1	1273.2	564.7	602.1	210.6	0.0	956.3	3.3	105.8	197.1
**Sum total**	1917.4	6545.2	3635.9	3762.3	939.1	0.0	2169.9	267.3	581.7	648.0

For abbreviations and structures of HMOs, please refer to Table S1, Supporting Information

NA, not determined.

### 3.2 Fecal Microbiota Composition and Microbial Clusters

Illumina HiSeq sequencing of the V4 region of bacterial 16S rRNA genes yielded 14 474 685 high‐quality reads that passed the quality check and could be assigned to 531 OTUs from 113 genera. In case an OTU could not be classified to a given genus level, it was assigned to the next available taxonomic rank. The predominating genera in this infant cohort were *Bifidobacterium, Bacteroides, Escherichia–Shigella*, and *Parabacteroides*, with a mean relative abundance of 32% (0–91.5%), 21.3% (0–76.7%), 11.8% (0–57.8%), and 6.7% (0–64%), respectively. Genera with the highest number of contributing OTUs included *Bacteroides* (64 OTUs), *Bifidobacterium* (38 OTUs), *Parabacteroides* (31 OTUs), *Lactobacillus* (24 OTUs), and *Streptococcus* (20 OTUs). Eighty two OTUs of the total 531 OTUs were found in at least 6 or more infants of the 121 infants studied (Table S3, Supporting Information). The remaining low prevalence OTUs are found in five or fewer infants and are summarized as “Other” (Table S3, Supporting Information). Overall, the fecal microbiota composition of infants in this cohort was highly variable, yet we could distinguish presence of three universal microbial patterns based on DMM cluster analysis (Figure S1, Supporting Information). These clusters were characterized by microbial communities with a mixed structure (Cluster A), or by communities with either a high relative abundance of *Bifidobacterium* (Cluster C), or a high relative abundance of both *Bifidobacterium* and *Bacteroides* (Cluster B).

### 3.3 The Effect of HMOs in Breast Milk and Other Factors on Fecal Microbiota Composition

**Figure 1:**
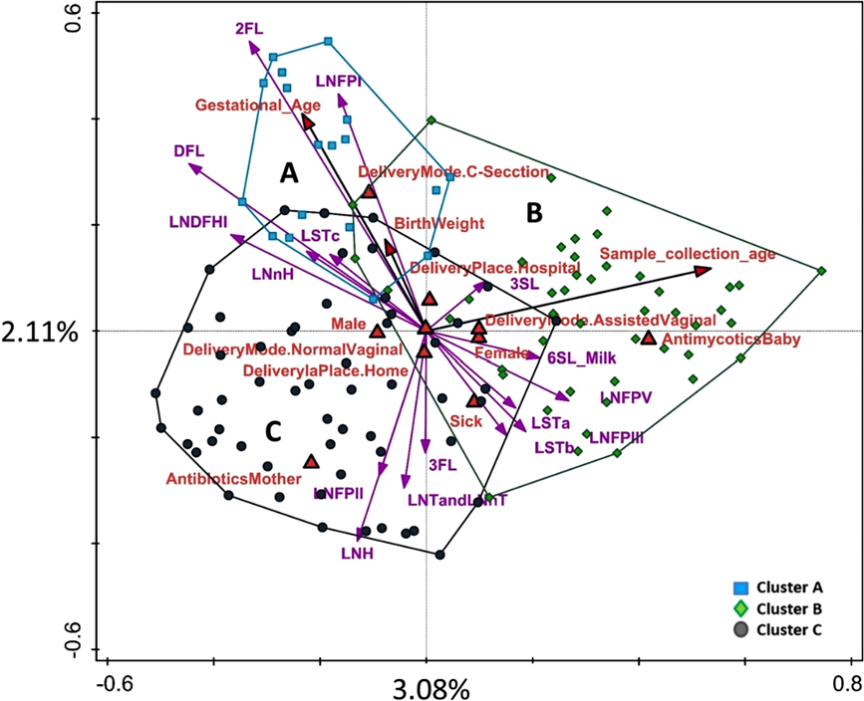
Constrained analysis (RDA) of different factors and milk HMO levels and their association with the fecal OTU profile of infants. Samples are labeled and enveloped based on the infant assignment to microbial cluster type A, B, or C.

We used RDA to identify factors affecting fecal microbiota composition of infants in the study (**Figure** 1). Together, the explanatory variables explained 21.7% of the variation in the OTU data. However, only mode of delivery and gender had a significant effect on microbiota composition (*p* < 0.05), and milk 2′FL concentration was borderline significant (*p* = 0.06). PLS regression also showed a significant association between 2′FL (and LNFPI) concentrations in milk with infant microbiota (FDR <0.05, Table S4, Supporting Information). When samples were color‐coded by infant's DMM cluster type, we also noted that, based on the RDA vector distribution, high levels of 2′FL and LNFPI in milk, as well as C‐section, were all associated with microbial cluster A, which was characterized by a mixed microbial profile (Figure 1). The association between breast milk HMO composition and the assignment of each infant to a specific microbial cluster type was further investigated using RDA (data not shown). The results indicated an association between overall HMO composition in milk and the DMM cluster type (*p* < 0.05, 3.1% explained). We used Chi‐square analysis to test if the mother's secretor status (yes/no), which is known to affect the ability to synthesize (α1‐2)‐linked fucose, and thus, the amount of some major neutral HMOs in milk (e.g., 2′FL and LNFPI), had an effect on infant microbiota profiles, as characterized by different DMM cluster types, but the association was not significant (*p* = 0.08) (Table S5, Supporting Information). Also, infant gender was not significantly associated with any of the DMM cluster types.

### 3.4 The Association Between Infant Fecal Microbiota Composition and HMO Degradation

**Figure 2:**
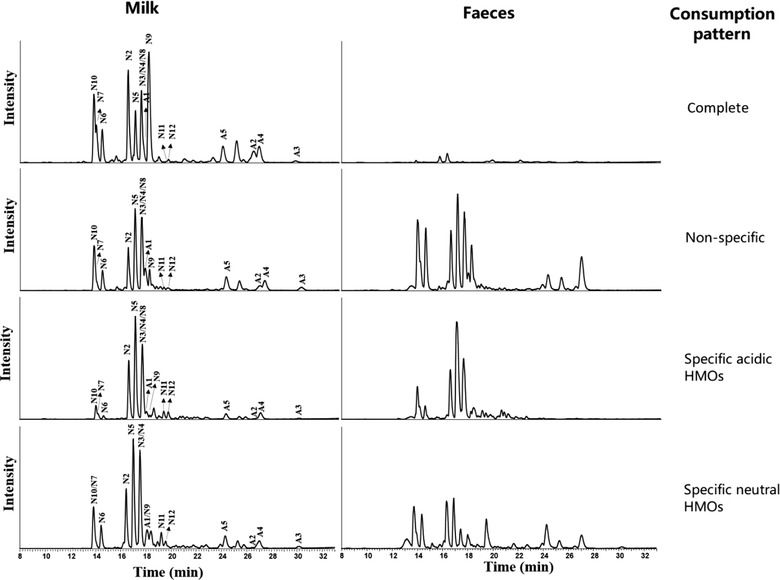
Different utilization patterns of infants defined based on the comparison of HMO profiles in breast milk and infant feces. Peak assignments are as follows: N2‐2′FL, N3‐LNT, N4‐LNnT, N5‐LNFP I, N6‐LNFP II, N7‐LNFP III, N8‐LNFP V, N9‐DFL, N10‐LNDFH I, N11‐LNH, N12‐LNnH; S1‐6′SL, S2‐3′SL, S3‐LSTa, S4‐LSTb, S5‐LSTc.

By comparing the HMO profiles in breast milk and in corresponding infant feces, we detected the presence of five patterns in the HMO consumption (**Figure** 2). The first pattern (“Complete”) was characterized by low or undetectable amounts of any of the HMOs in infant feces, suggesting a complete consumption of all HMOs from the breast milk. The second pattern (“Non‐specific”) showed a fecal HMO profile that was comparable to that of breast milk and contained high concentrations, thus implying a non‐specific (or broad) and incomplete (or slow) consumption of HMOs by the infant GI tract microbiota. The third pattern (“Specific”) indicated selective consumption of specific HMOs and was further divided into three types. “Specific acidic” showed a high level of neutral HMOs in feces, meaning that the acidic HMOs (3′SL, 6′SL, LSTa, LSTb, LSTc) were predominantly utilized. “Specific neutral” was characterized by the acidic HMO profile of the feces, meaning that neutral HMOs were predominantly utilized by the infant GI tract microbiota. The third type was “Specific other,” which could not be categorized as neither acidic nor neutral HMOs (data not shown).

**Figure 3:**
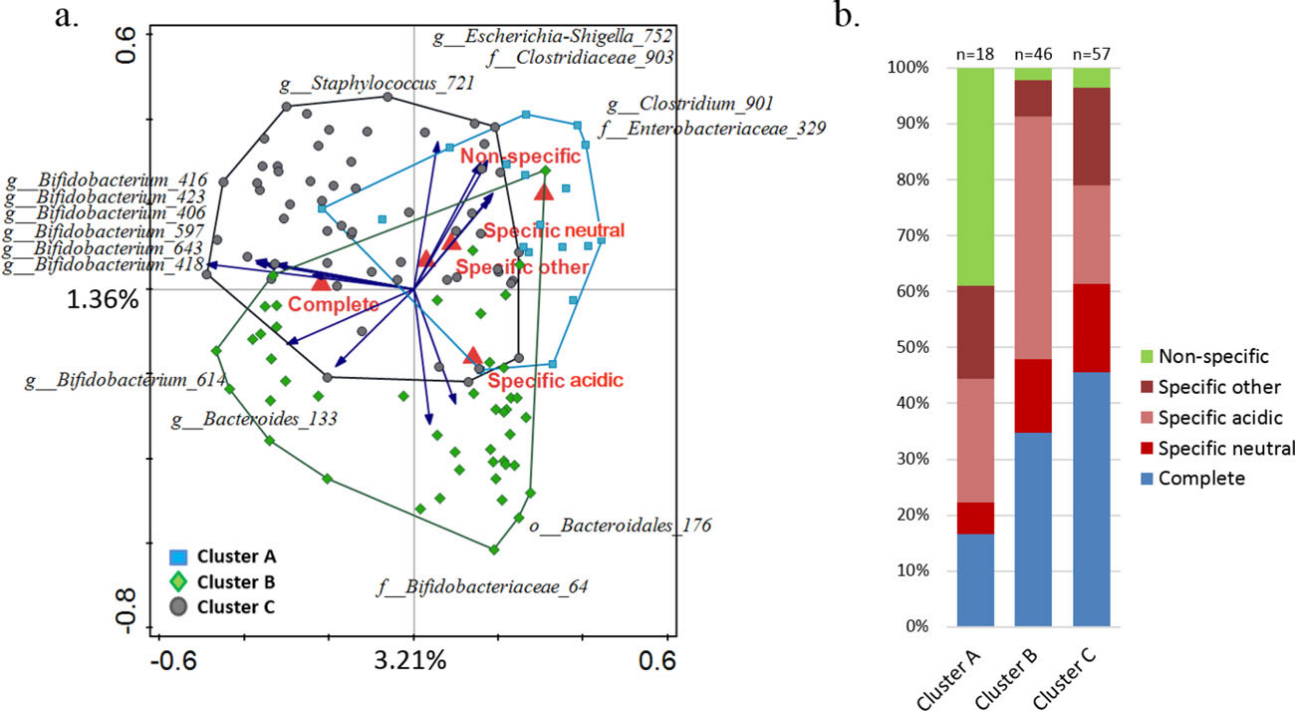
Association of general HMO consumption patterns with microbial cluster types A, B, C. a) RDA showing the association between HMO consumption patterns and microbial OTUs. Fifteen best fitting OTUs are displayed and samples are color‐coded based on their cluster type assignment; b) segregation of infants based on their HMO consumption pattern in relation to their microbial cluster type classification.

We used RDA analysis to investigate the association between microbiota composition and different HMO consumption patterns. We noted that “Complete,” “Non‐specific,” and “Specific acidic” consumptions were significantly associated with infant microbiota composition (FDR <0.05), while the association of “Specific neutral” and “Specific other” was not significant. In addition, “Complete” consumption correlated with high relative abundance of bifidobacteria, including the two highly abundant *Bifidobacterium* OTUs 614 and 418 (**Figure** 3a). Furthermore, the Chi‐square test showed a strong and significant association (χ^2^ = 32.28; *p* < 0.00001) between the frequency of different consumption patterns and each DMM microbial cluster. Forty percent of infants who were classified in the mixed microbial cluster A also showed a non‐specific HMO consumption pattern (Figure 3b).

**Figure 4:**
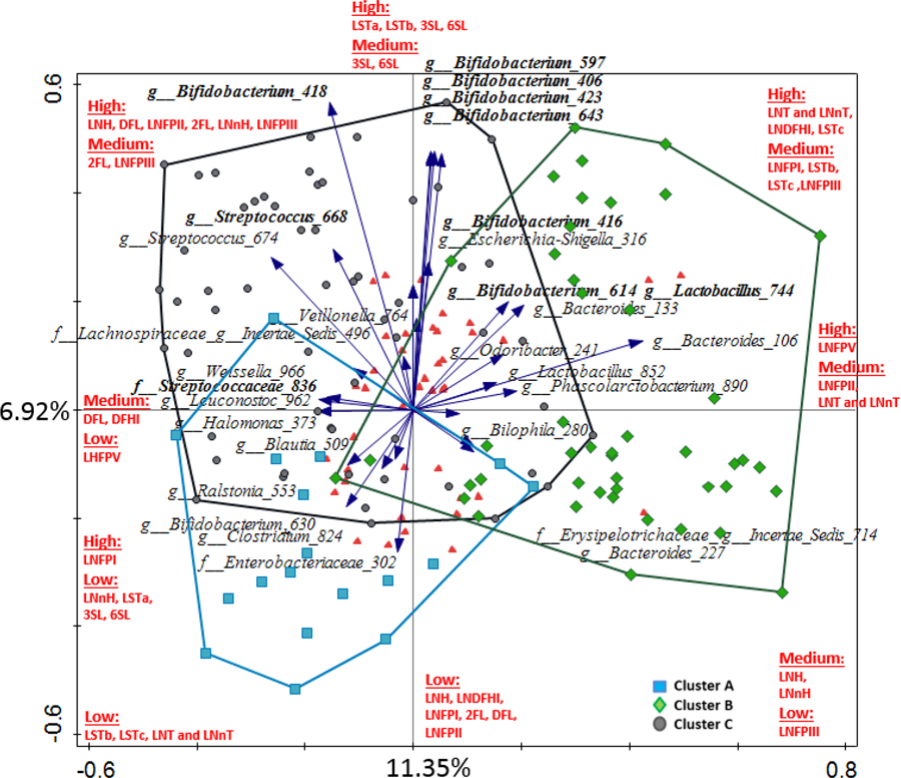
RDA showing the association between the degree of degradation of individual HMOs and microbial OTUs. OTUs which were significantly (*p* < 0.05) increased in high‐degrading infants for at least one of the HMOs are displayed. Taxa with FDR <0.05 are highlighted in bold. For more information on average relative abundance of the displayed OTUs in the study population and the detailed results of Kruskal–Wallis analyses (see Tables S3 and S6, Supporting Information). Samples are color‐coded based on microbiota cluster type assignment. Red triangles indicate consumption of each HMO, as summarized in red text.

**Figure 5:**
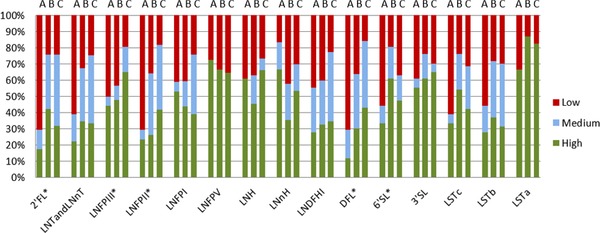
Proportion of infants showing either “high,” “medium,” or “low” HMO consumption levels, within each microbial cluster class A, B, C. Significant differences in distribution as determined by Chi‐square analysis are indicated with an asterisk.

In order to investigate the association between microbiota composition and consumption of specific HMOs in more depth, we classified infants as “low,” “medium,” or “high” consumers for each of the measured HMOs. We then used this classification in the multivariate RDA analysis and showed that the HMO consumption explained 61.5% of variation in microbiota. Infant's degradation ability of 2′FL, LNT and LNnT, DFL, 6′SL, LNH, LNFPII, LNFPIII (FDR <0.05), LSTb (FDR = 0.06), LSTc, and 3′SL (FDR = 0.07) was associated with differences in the fecal microbiota composition (**Figure** 4). For all HMO types, there was a general trend relating consumption efficiency and infant fecal microbiota cluster class. RDA showed that microbial DMM cluster type alone could explain 8.4% of variation in the consumption category and that the cluster effect was statistically significant (FDR <0.05). The lowest efficiency of consumption was linked to microbial cluster type A, with 40.6% at “high” level, 10% at “medium” level, and 49.4% of all HMOs consumed at “low” level. Infants classified in microbial cluster B showed high HMO consumption levels, with 47.3% of all HMO types consumed at “high” level, 21.3% consumed at “medium” level, and 31.4% at “low” level. Infants classified in microbial cluster C showed “high” consumption for 49.8% of all HMOs, “medium” consumption for 24.4%, and “low” consumption for 25.8%. The microbial cluster type consumption efficiency pattern varied for different HMO types (**Figure** 5). Chi‐square analysis was used to test the correlation between the proportion of infants in “high,” “medium,” and “low” consumption categories for each HMO and the infant microbial cluster groups. Significant (*p* < 0.05) differences were detected between clusters with respect to consumption of 2′FL, LNFPIII, LNFPII, DFL, and 6′SL. For the aforementioned HMOs, the highest proportion of infants with the lowest ability to break down these HMOs was found in cluster A (Figure 5).

**Figure 6:**
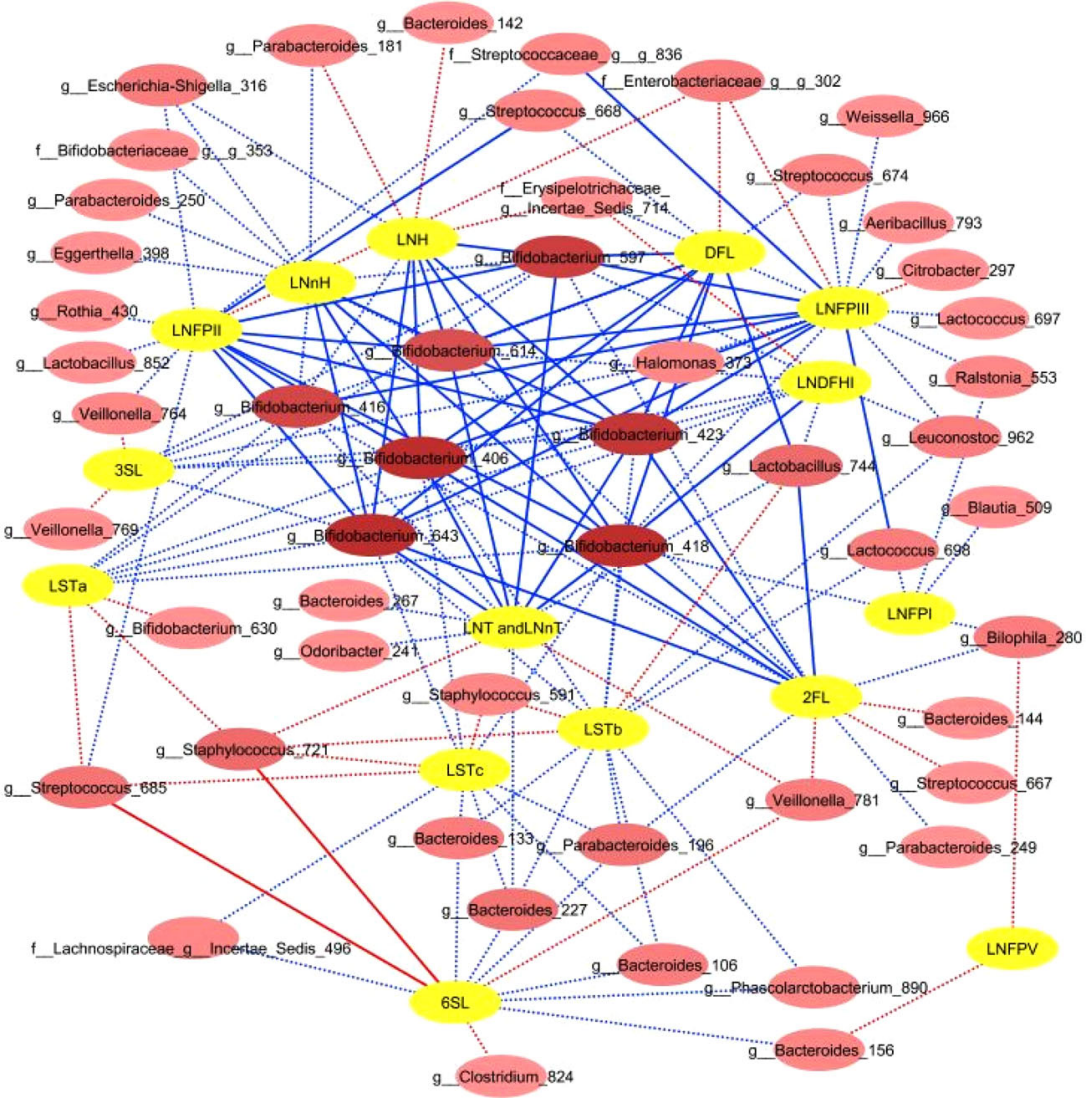
OTUs significantly associated with HMO consumption based on Kruskal–Wallis test including infants classified as high and low consumers for each HMO. Red lines indicate higher OTU relative abundance in relation to low HMO consumption; blue lines indicate higher relative abundance in relation to high consumption. OTU nodes which are connected with the highest number of HMOs are indicated by darker shades of pink. Dotted lines indicate associations with *p* < 0.05; solid lines indicate associations with FDR <0.05.

Kruskal–Wallis analysis was used to compare microbiota composition at the OTU level between infants who were classified as either “high” or “low” consumers for each HMO measured in this study (**Figure** 6 and Table S6, Supporting Information). Infants who showed “high” consumption of 2′FL and DFL had significantly higher relative abundance of OTUs *Bifidobacterium* 418 and *Lactobacillus* 744 (FDR < 0.05). In addition, “high” DFL consumption was associated with significantly higher relative abundance of *Bifidobacterium* OTUs 406, 643, 423, and 597. Similarly, infants who showed “high” consumption of LNT and LNnT, LNFPIII, LNFPII, and LNH had a significantly higher relative abundance of *Bifidobacterium* OTUs 418, 406, 643, 423, and 597. Relative abundance of *Bifidobacterium* 418 was higher in infants who were efficient degraders of LNnH and LNDFHI, and *Bifidobacterium* 416 was associated with degradation of LNFPII, and *Bifidobacterium* 614 with LNFPII and LNH. We could not detect statistically significant differences (with FDR <0.05) in the relative abundance of taxa between infants characterized as “high” and “low” degraders of LNFPI, LNFPV, 3′SL, LSTa, LSTb, and LSTc.

Kruskal–Wallis test comparing infants who were classified as “high” and “medium” consumers for different HMOs showed no statistically significant differences in distribution of any of the OTUs (data not shown). Infants classified as “low” and “medium” consumers showed significant differences (FDR <0.05) in relative abundance of *Bifidobacterium* 418 for 2′FL and LNFPII; *Bifidobacterium* OTUs 418, 643, 406, and 423 for DFL; *Bifidobacterium* 418 and *Bifidobacterium* 416 for LNFPIII; *Bifidobacterium* OTUs 418, 643, 406, 597, and 423 for LNT and LNnT (data not shown). The observed patterns in the abundance of these taxa between the “low” and “medium” consumption classes mimicked those seen between “low” and “high” consumers for these HMOs (Table S6, Supporting Information).

## 4 Discussion

Fecal microbiota composition of healthy, 1‐month‐old breastfed infants was characterized in this study using 16S rRNA gene sequencing and revealed high inter‐individual variability in the fecal microbiota composition. Microbial patterns could be observed in the sequencing data, and the DMM analysis revealed that all infants could in fact be classified into three categories based on their fecal microbial profiles. Even though the high variability in infant fecal microbiota composition had been reported,^31^ the occurrence of similar microbial clusters had been described previously in only one study.^32^ The origin of these microbial patterns seen during infancy and their health implications are still unknown.

Several factors in early life may affect the dynamics of the developing infant GI microbiota. Our results showed that at about 4 weeks of age, mode of delivery and gender could explain the observed variation in the microbiota composition. The effect of mode of delivery has been indicated in infants of similar age in another study.^33^ Also, the effect of gender in infants of that age had been implied previously using qPCR/RTqPCR analyses for specific species/strain detection in feces.^34^ Our study shows that gender‐associated differences can also be detected at the microbial community level in healthy breastfed infants.

### 4.1 Breast Milk HMO Levels Have a Limited Effect on Fecal Microbiota Composition in 1‐Month‐Old Infants

One of the key factors shaping infant microbiota in early life is infant diet. Exclusively breastfed infants show high inter‐individual variability, leading to the question whether these differences can be linked to the breast milk properties, including the unique HMO composition of the milk. It has been estimated that in healthy breastfed infants most of the ingested HMOs can reach the colon undigested.^35^, ^36^, ^37^ These HMOs serve as an abundant and diverse carbon source available for bacterial fermentation.^5^ Using PLS modeling, we could detect statistically significant associations between infant fecal microbiota composition and milk LNFPI and 2′FL levels (Table S4, Supporting Information). Both, LNFPI and 2′FL are neutral, fucosylated, unbranched HMOs with an α1,2 linkage joining fucose and galactose, and they were on average the third and fourth most abundant HMO measured in our dataset. The same association was found in another study of 3‐month‐old breastfed infants, albeit using a much smaller cohort (*n* = 16), which further indicated that LNFPI was positively associated with *Bacteroides* and *Bifidobacterium*, and 2′FL with *Bacteroides*.^7^ Based on our RDA analysis (Figure 1), these two HMOs were associated with mixed microbiota cluster type A, which is characterized by low relative abundance of *Bacteroides* and *Bifidobacterium*. In addition, none of the other HMOs showed significant association with microbiota composition, but based on the vector positions (Figure 1), one could argue that higher concentrations of a number of different HMOs in maternal milk could be driving the infant microbiota away from cluster A, but not specifically towards clusters B or C. Some of the possible explanations could be that a combined effect of a number of HMO structures may be necessary to guide microbiota development in early life, or that stronger associations develop over a longer period, and that at 1 month of age the microbial profile of infants in our study was still largely in its transitional phase.^38^, ^39^ In addition, other HMO structures that were not measured in our study might also play a role, as well as the undigested residual lactose from breast milk, which in the lower intestine could also be readily fermented by the resident microbiota.

Other breast milk components, such as secretory IgA, lactoferrin, lysozyme, as well as the breast milk microbiota itself, are all likely to contribute to shaping the structure of microbial communities within infants’ GI tracts^2^, ^40^, ^41^, ^42^, ^43^ and possibly concealing the effect of the individual HMOs. Over 200 different microbial genera had been identified in human milk to date, of which *Streptococcus* and *Staphylococcus* tend to be the most predominant taxa.^44^, ^45^, ^46^ Vertical transfer between mother's breast milk and infant's GI tract of viable populations of different microbial groups, including few species and strains of *Lactobacillus* and *Bifidobacterium* had also been confirmed and implied as one of the key factors in establishing infant's gut microbiota.^46^ Breast milk also contains large amounts of lysozyme, up to 400 µg mL^−1^, which acts selectively on different microbial species; for example, both *Bifidobacterium bifidum* and *Bifidobacterium longum* had been shown to be resistant to lysozyme, whereas clostridia, and many other Gram‐positive and Gram‐negative bacteria were highly susceptible.^43^ Thus, the modulatory function of breast milk likely comes from the synergetic effect of all of its components, and HMOs alone might not be the driving factor in shaping the microbiota in early life.

### 4.2 HMO Consumption Patterns are Associated with Specific Microbial Groups

The matched analysis of HMO profiles from breast milk and fecal samples allowed us to estimate the in vivo HMO degradation levels within each infant, and to study in much greater detail the relationship between infant GI microbiota composition and HMO utilization. Based on this novel approach, we were able to classify infants into five HMO consumption groups. A previous pilot study reported only two types of HMO fecal profiles in infants, namely the neutral and the acidic profiles.^47^ However, links between these utilization patterns and the infant fecal microbiota composition have remained largely unknown. Our data showed a strong significant association between “Non‐specific” (or low) consumption and the microbial DMM cluster A, whereas the “Complete” consumption pattern was related with cluster C and “Specific‐acidic” with cluster B (Figure 3). Thus, even though the GI tract microbial ecosystem is still in its early establishment phase at 1 month of age, we showed that the degradation of different types of HMOs was carried out by specific bacterial assemblages, with evolved mechanisms for efficient consumption of these abundant food components in milk.

Bifidobacteria are the main group of microorganisms in the lower GI tract of healthy infants, and also the main consumers of HMOs.^48^, ^49^, ^50^ Our data support this, as the most abundant and prevalent OTUs detected were of bifidobacterial origin (Table S3, Supporting Information). Earlier in vitro studies showed highly specific metabolic behavior of different bifidobacterial species and strains with respect to their ability to utilize different HMOs.^6^ The most prevalent OTU in our set was *Bifidobacterium* 614, with an average relative abundance of 23.3% and prevalence of 92%. NCBI blast analysis revealed that the OTU sequence (Table S7, Supporting Information) matched several different species and strains of *Bifidobacterium*, including various strains of *B. longum* (*infantis*), commonly found in the infant GI tract. Our analysis showed that *Bifidobacterium* 614 was associated with high in vivo consumption of various HMOs, specifically 2′FL, DFL, LNDFHI, LNFPII, LNFPIII, LNH, and LNT and LNnT (Table S6, Supporting Information). The second most abundant OTU was *Bifidobacterium* 418, found in 45% of infants, with an average relative abundance of 6.6%. The NCBI blast analysis of OTU 418 returned a 100% match to several different strains of *B. bifidum* (DSM 20456 = ATCC 29521 = JCM 1255, NBRC100015, KCTC3202). The presence of *Bifidobacterium* 418 correlated strongly (FDR <0.05) with high consumption levels of 2′FL, LNT and LNnT, LNFPIII, LNFPII, LNH and its isomer LNnH, LNDFHI, and DFL, and with LNFPI, LSTa, LSTb, and LSTc (*p* < 0.05). *B. bifidum* has been shown to be an efficient HMO degrader in in vitro fermentation studies able to secrete glycosidases to degrade HMOs extracellularly, also making it possible for other species/subspecies to access the HMO degradation byproducts and metabolites during cross‐feeding.^6^ In vitro studies showed that *B. bifidum* DSM 20456 could efficiently degrade LNT, 2′FL, LNnT, LNFPI, LNFPII, LNFPIII, and LNDFHI, though the rate at which it was degrading these HMOs varied.^6^

*Streptococcus* and *Staphylococcus* OTUs showed an increase in relative abundance specifically in relation to high consumption of the fucosylated HMOs—DFL, LNFPII, and LNFPIII. Although, in vitro studies showed that *Streptococcus* and *Staphylococcus* cannot effectively metabolize HMOs,^51^ it has been shown that the presence of HMOs may enhance growth of breast milk–associated *Staphylococcus* by activating growth‐promoting signaling and without being actively metabolized by this strain.^51^ *Streptococcus* and *Staphylococcus* cross‐feeding on HMO metabolites might also play a role, though to date there are no studies documenting it.

*Bacteroides* and *Parabacteroides* (formerly also *Bacteroides*) are among the first dominant bacterial groups, next to bifidobacteria, established in the infant GI tract.^52^ In general, members of the genus *Bacteroides* can degrade a broad range of simple and complex sugars, oligosaccharides, and polysaccharides, including HMOs, mucus glycans, and plant‐derived polysaccharides.^3^ Like bifidobacteria, *Bacteroides* spp. can grow on milk glycans as a sole carbon source; however, bifidobacteria might be better adapted to utilize a wider range of HMO structures, including simple HMO structures, as it has been shown for *B. infantis* and LNnT.^52^ The trophic niche overlap might explain why infants with high levels of *Bifidobacterium*, such as those classified in the DMM cluster C tend to have lower levels of *Bacteroides*. On the other hand, *Bacteroides* has been shown to efficiently degrade mucus glycans, and because of the similarity of HMO structures and mucus glycans, some *Bacteroides* species could also effectively degrade specific HMOs by activating the mucus‐degrading pathway.^52^, ^53^ These species might be better at competing with bifidobacterial groups, especially those species of *Bifidobacterium* which might be less adapted for HMO utilization. Our analysis indicated that infants who were efficient degraders of the sialylated (acidic) HMOs (3′SL, 6′SL, LSTa, LSTb, LSTc) and classified into “Specific acidic” consumption category were also often assigned to the *Bacteroides* dominated DMM cluster B (Figure 2). This was in agreement with another study which showed that among others, the HMOs 3′SL and 6′SL could be used as sole carbon source to support growth of *Bacteroides fragilis, Bacteroides vulgatus*, and *Bacteroides thetaiotaomicron*.^54^ Furthermore, these species, as well as few other species, including certain strains of *B. longum* were also shown to metabolize sialic acid.^55^

Finally, a biologically important microbial group commonly detected in infant feces is the lactobacilli. Our results show that high levels of degradation of 2′FL, DFL, LNDFHI, LNT and LNnT, and LNFPII were significantly correlated with higher relative abundance of this group. Remarkably, the opposite effect was noted for LSTb (Figure 6). Unfortunately, the two interesting lactobacilli OTUs which were identified in our data, namely *Lactobacillus* 744 and *Lactobacillus* 852, had sequence reads which returned a 100% match to more than a dozen species and strains of lactobacilli in the NCBI blast analysis, making it impossible to unequivocally identify these populations to the species level. Several *Lactobacillus* spp. have been frequently isolated from neonate feces, including *L. fermentum, L. casei, L. paracasei, L. delbrueckii, L. gasseri, L. rhamnosus*, and *L. plantarum*.^56^ These lactobacilli were shown to be unable to efficiently ferment HMOs in vitro^57^, ^58^; however, they have been shown to grow well on HMO metabolites in vitro.^58^ Thus, via the cross‐feeding with other bacteria, for example, bifidobacteria, it is possible that HMO degradation can be linked with higher relative abundance of lactobacilli and other community members in the microbial ecosystem within GI tract.

The roles of different HMOs in the development of infant GI tract microbiota, the occurrence of microbial clusters, and the nutritional and health consequences relating to the existence of different trophic networks that are built upon the degradation of specific HMOs are still mostly unknown. Our results confirmed the central role of bifidobacteria in the HMO breakdown and provided an insight into different microbial assemblages in healthy, 1‐month‐old infants. Furthermore, carrying out the analyses at the OTU level allowed us to uncover a higher level of detail showing that, for example, bifidobacteria were associated with both clusters B and C (17% and 41%, respectively), but the distribution of specific bifidobacterial OTUs within these two clusters was not identical (data not shown). Until now, few in vitro studies demonstrated that closely related species or strains might exhibit different metabolic activities and be involved in a range of complementary trophic interactions. Future studies should strive to identify the species or strains that are present in the infant gut and to build understanding on the interactions between these species. In the future, a better understanding on how the bacterial assemblages form in vivo and the identification of the key species and their roles in driving the colonization, as well as their effects on the host, could be translated into practical applications within infant nutrition and health.

## 5 Conclusion

GI tract microbiota composition in 1‐month‐old breastfed infants is shaped by multiple factors, including HMOs. We observed a direct link between 2′FL and LNFP I in breast milk and microbial community composition in this cohort, but it is likely that the infant microbiota is shaped through the combined effect of all HMOs and other bioactive components in breast milk. We showed that breast milk HMO degradation patterns differed among infants belonging to different microbial cluster types. Degradation of specific HMOs could be correlated with an increase in relative abundance of various phylotypes (OTUs) within the genus *Bifidobacterium* and to a lesser extent within the genera *Bacteroides* and *Lactobacillus*.

## Conflict of Interest

The authors declare no conflict of interest.

## Supporting information

Supporting Information
