# Supplementary material for: Correlating Infant Fecal Microbiota Composition and Human Milk Oligosaccharide Consumption by Microbiota of 1‐Month‐Old Breastfed Infants
Source: Mol Nutr Food Res. 2019 Apr 30;63(13):1801214. doi: 10.1002/mnfr.201801214 (PMC6618098; doi:10.1002/mnfr.201801214)
Supplement: Supplementary file 1 — Supporting Information [file MNFR-63-na-s001.docx]

**Supplementary Figures and Tables**


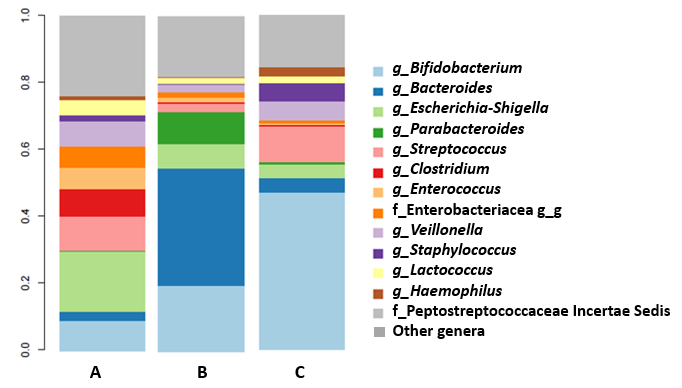


Figure S1. Microbial cluster composition based on DMM modelling of KOALA samples

Table S1. HMO categories, names and structures [50] included in this study. Blue circle – glucose; yellow circle – galactose; blue square N – acetylglucosamine; red triangle – fucose; purple diamond – sialic acid

| Category | Name | Abbreviation | Structure |
| --- | --- | --- | --- |
| Neutral | 3-Fucosyllactose | 3FL | 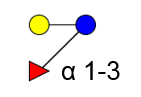 |
|  | 2'-Fucosyllactose | 2'FL | 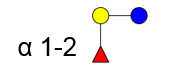 |
|  | Lacto-*N*-tetraose | LNT | 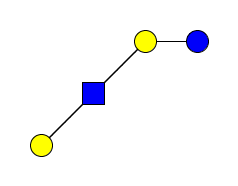 |
|  | Lacto-*N*-neotetraose | LNnT | 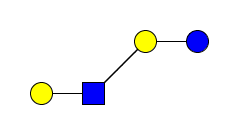 |
|  | Lacto-*N*-fucopentaose I | LNFP I | 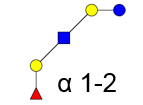 |
|  | Lacto-*N*-fucopentaose II | LNFP II | 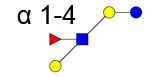 |
|  | Lacto-*N*-fucopentaose III | LNFP III | 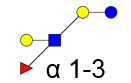 |
|  | Lacto-*N*-fucopentaose V | LNFP V | 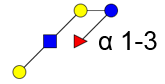 |
|  | Difucosyllactose | DFL | 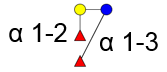 |
|  | Lacto-*N*-difucohexaose I | LNDFH I | 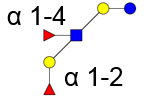 |
|  | Lacto-*N*-hexaose | LNH | 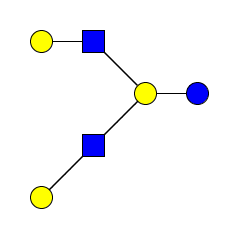 |
|  | Lacto-*N*-neohexaose | LNnH | 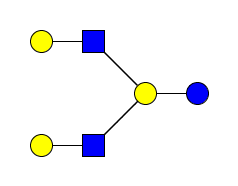 |
| Acidic | 6'-Sialyllactose | 6'SL | 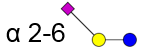 |
|  | 3'-Sialyllactose | 3'SL | 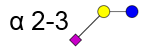 |
|  | Sialyl-lacto-*N*-tetraose a | LST a | 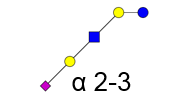 |
|  | Sialyl-lacto-*N*-tetraose b | LST b | 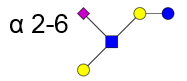 |
|  | Sialyl-lacto-*N*-tetraose c | LST c | 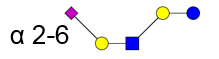 |

Table S2. Infant – mother pair demographics

| **Infant/Mother pairs (n=121)** | | **n** |
| --- | --- | --- |
| Delivery mode | Normal Vaginal  Assisted Vaginal  C-Section | 100  11  10 |
| Delivery Place | Home  Hospital | 72  49 |
| Gender | Female  Male | 59  62 |
| Gestation (weeks) | Mean ± SEM  Minimum  Maximum | 40.24 ±1.16  37.14  42.86 |
| Birth Weight (g) | Mean ± SEM  Minimum  Maximum | 3651 ±43.48  2140  4780 |
| Age at collection (days) | Mean ± SEM  Minimum  Maximum | 32.56 ±0.5  24  56 |
| Health at collection | Sick  Not Sick | 3  118 |
| Medication Use at time of collection | Antibiotics (Baby)  Antimycotics (Baby)  Antibiotics (Mother) | 0  2  2 |

Table S3. Average relative abundance and prevalence of the OTUs shared by at least 5% of infants in this study.

| **OTU** | **Average RA (%)** | **Prevalence (%)** |  | **OTU** | **Average RA (%)** | **Prevalence (%)** |
| --- | --- | --- | --- | --- | --- | --- |
| f_Bifidobacteriaceae_64_g_g | 0.03 | 11 |  | *g_Lactococcus_698* | 0.35 | 50 |
| f_Bifidobacteriaceae_353_g_g | 0.01 | 7 |  | *g_Lactococcus_697* | 0.03 | 10 |
| *g_Bifidobacterium_614* | 23.32 | 92 |  | *g_Lactococcus_696* | 0.01 | 6 |
| *g_Bifidobacterium_418* | 6.64 | 45 |  | f_Enterobacteriaceae_302_g_g | 3.83 | 31 |
| *g_Bifidobacterium_622* | 1.33 | 50 |  | *g_Escherichia-Shigella_328* | 11.70 | 72 |
| *g_Bifidobacterium_643* | 0.13 | 29 |  | *g_Escherichia-Shigella_316* | 0.04 | 17 |
| *g_Bifidobacterium_406* | 0.13 | 29 |  | *g_Staphylococcus_721* | 2.08 | 58 |
| *g_Bifidobacterium_423* | 0.12 | 27 |  | *g_Staphylococcus_591* | 0.02 | 7 |
| *g_Bifidobacterium_597* | 0.11 | 26 |  | *g_Veillonella_781* | 0.83 | 48 |
| *g_Bifidobacterium_416* | 0.03 | 16 |  | *g_Veillonella_769* | 0.50 | 20 |
| *g_Bifidobacterium_630* | 0.02 | 9 |  | *g_Veillonella_764* | 0.27 | 22 |
| *g_Bifidobacterium_356* | 0.02 | 7 |  | *g_Veillonella_776* | 0.09 | 10 |
| Other *g_Bifidobacterium* (n=26) | 0.12 | 26 |  | *g_Haemophilus_368* | 0.88 | 49 |
| *g_Bacteroides_106* | 8.61 | 59 |  | *g_Clostridium_824* | 0.85 | 7 |
| *g_Bacteroides_149* | 4.58 | 22 |  | *g_Clostridium_885* | 0.48 | 9 |
| *g_Bacteroides_144* | 2.04 | 24 |  | *g_Blautia_509* | 0.47 | 7 |
| *g_Bacteroides_159* | 1.94 | 23 |  | *g_Blautia_471* | 0.04 | 5 |
| *g_Bacteroides_157* | 0.78 | 25 |  | *g_Phascolarctobacterium_890* | 0.21 | 7 |
| *g_Bacteroides_125* | 0.60 | 26 |  | *g_Phascolarctobacterium_892* | 0.12 | 5 |
| *g_Bacteroides_133* | 0.49 | 20 |  | f_Lachnospiraceae *Incertae_Sedis*_487 | 0.77 | 9 |
| *g_Bacteroides_156* | 0.33 | 12 |  | f_Lachnospiraceae *Incertae_Sedis*_496 | 0.05 | 5 |
| *g_Bacteroides_126* | 0.30 | 20 |  | f_Lachnospiraceae *Incertae_Sedis*_941 | 0.19 | 7 |
| *g_Bacteroides_142* | 0.20 | 6 |  | *g_Rothia_430* | 0.16 | 22 |
| *g_Bacteroides_139* | 0.08 | 5 |  | *g_Sutterella_565* | 0.79 | 21 |
| *g_Bacteroides_88* | 0.08 | 5 |  | *g_Leuconostoc_962* | 0.06 | 19 |
| *g_Bacteroides_267* | 0.05 | 5 |  | *g_Enterococcus_842* | 0.20 | 18 |
| *g_Bacteroides_227* | 0.02 | 5 |  | *g_Halomonas_373* | 0.06 | 18 |
| Other *g_Bacteroides* (n=50) | 1.26 | 27 |  | *g_Haemophilus_371* | 0.08 | 17 |
| *g_Parabacteroides_181* | 3.29 | 37 |  | *g_Bilophila_280* | 0.12 | 13 |
| *g_Parabacteroides_179* | 2.00 | 18 |  | *g_Flavonifractor_737* | 0.33 | 11 |
| *g_Parabacteroides_249* | 0.53 | 19 |  | *g_Aeribacillus_793* | 0.04 | 10 |
| *g_Parabacteroides_250* | 0.05 | 5 |  | *g_Collinsella_528* | 0.10 | 10 |
| *g_Parabacteroides_196* | 0.02 | 6 |  | *g_Eggerthella_398* | 0.02 | 7 |
| Other_*g_Parabacteroides* (n=25) | 0.79 | 22 |  | *g_Ralstonia_553* | 0.02 | 7 |
| *g_Lactobacillus_852* | 1.03 | 26 |  | *g_Weissella_966* | 0.01 | 7 |
| *g_Lactobacillus_744* | 0.78 | 36 |  | *g_Odoribacter_241* | 0.10 | 6 |
| Other_g_Lactobacillus (n=22) | 0.48 | 12 |  | *g_Citrobacter_297* | 0.02 | 5 |
| f_Streptococcaceae_836_g_g | 0.06 | 18 |  | *g_Negativicoccus_759* | 0.02 | 5 |
| *g_Streptococcus_685* | 2.46 | 74 |  | *g_Varibaculum_438* | 0.02 | 5 |
| *g_Streptococcus_668* | 0.18 | 19 |  | f_Erysipelotrichaceae *Incertae_Sedis*_714 | 0.69 | 7 |
| *g_Streptococcus_674* | 0.16 | 36 |  | f_Ruminococcaceae_6_g_g | 0.31 | 6 |
| *g_Streptococcus_684* | 0.15 | 6 |  | f_Coriobacteriaceae_929 | 0.03 | 8 |
| *g_Streptococcus_667* | 0.13 | 12 |  | f_Peptostreptococcaceae *Incertae_Sedis*_394 | 0.02 | 9 |
| Other_*g_Streptococcus* (n=14) | 0.67 | 13 |  | Remaining Other OTUs (n=312) | 6.81 | 87 |
|  |  |  |  |  |  |  |
| Total (n=531) | 100.0 | 100 |  |  |  |  |

Table S4. PLS analysis results showing the association of milk and faecal HMOs with microbiota of 121 infants. p<0.05 highlighted in bold.

|  | **MILK** |  | **FAECES** |  |
| --- | --- | --- | --- | --- |
| Compound | R2 | p-value | R2 | p-value |
| 2´FL | 0.449014 | **0.01** | 0.474618 | **0.008** |
| LNT and LNnT | 0.365672 | 0.321 | 0.486463 | **0.005** |
| LNFPIII | 0.411369 | 0.1 | 0.334021 | 0.489 |
| LNFPII | 0.428876 | 0.059 | 0.538998 | **0.001** |
| LNFPI | 0.453009 | **0.006** | 0.352368 | 0.395 |
| LNFPV | 0.361059 | 0.29 | 0.411458 | 0.268 |
| LNH | 0.296281 | 0.711 | 0.419855 | 0.311 |
| LNnH | 0.372147 | 0.267 | 0.407214 | 0.372 |
| LNDFHI | 0.348777 | 0.378 | 0.425054 | **0.02** |
| DFL | 0.375545 | 0.193 | 0.484093 | **0.001** |
| 6´SL | 0.308867 | 0.679 | 0.307907 | 0.651 |
| 3´SL | 0.330597 | 0.538 | 0.335586 | 0.548 |
| LSTc | 0.40498 | 0.101 | 0.284052 | 0.797 |
| LSTb | 0.3488 | 0.387 | 0.366729 | 0.303 |
| LSTa | 0.37228 | 0.267 | 0.303566 | 0.667 |

Table S5. Chi-square analysis results. The is no association between mother secretor status and infant microbial DMM cluster type (χ^2^ (2) = 5.13; p=0.08)

|  | **Secretor +** | | | **Secretor -** | | | **Totals** |
| --- | --- | --- | --- | --- | --- | --- | --- |
| **Cluster** | Observed | Expected | χ^2^ | Observed | Expected | χ^2^ |  |
| A | 31 | 33.45 | 0.18 | 15 | 12.55 | 0.48 | 46 |
| B | 17 | 13.09 | 0.17 | 1 | 4.91 | 3.11 | 18 |
| C | 40 | 41.45 | 0.05 | 17 | 15.55 | 0.14 | 57 |
| **Totals** | 88 |  |  | 33 |  |  | 121 |

Table S6. OTUs significantly different (p<0.05) in relative abundance between infants classified as high or low consumers of specific HMOs. Differences in relative abundance with FDR<0.05 are indicated in bold.

| **Taxonomy** | **OTU** | **HMO** | **p** | **FDR** | **High consumption group** | **Low consumption group** |
| --- | --- | --- | --- | --- | --- | --- |
| *g_Bacteroides* | 106 | 6´SL | 0.008 | 0.137 | 0.1021 | 0.0355 |
|  | 106 | LSTb | 0.010 | 0.192 | 0.0745 | 0.0279 |
|  | 106 | LSTc | 0.006 | 0.129 | 0.1047 | 0.0288 |
|  | 133 | 6´SL | 0.005 | 0.136 | 0.0079 | 0.0002 |
|  | 133 | LSTb | 0.005 | 0.131 | 0.0078 | 0.0003 |
|  | 133 | LSTc | 0.002 | 0.129 | 0.0087 | 0.0002 |
|  | 142 | LNH | 0.034 | 0.276 | 0.0001 | 0.0056 |
|  | 144 | 2´FL | 0.017 | 0.168 | 0.0269 | 0.0271 |
|  | 156 | 6´SL | 0.021 | 0.263 | 0.0020 | 0.0010 |
|  | 156 | LNFPV | 0.040 | 0.848 | 0.0005 | 0.0047 |
|  | 227 | 6´SL | 0.040 | 0.296 | 0.0004 | 0 |
|  | 227 | LNTandLNnT | 0.022 | 0.176 | 0.0004 | 0 |
|  | 227 | LSTb | 0.022 | 0.227 | 0.0006 | 0 |
|  | 227 | LSTc | 0.030 | 0.273 | 0.0004 | 0 |
|  | 267 | LNTandLNnT | 0.041 | 0.280 | 0.0011 | 0 |
| *g_Bifidobacterium* | **406** | **2´FL** | **0.001** | **0.018** | **0.0015** | **0.0002** |
|  | 406 | 3´SL | 0.033 | 0.410 | 0.0014 | 0.0005 |
|  | **406** | **DFL** | **0.000** | **0.001** | **0.0015** | **0** |
|  | 406 | LNDFHI | 0.002 | 0.059 | 0.0015 | 0.0004 |
|  | **406** | **LNFPII** | **0.000** | **0.000** | **0.0024** | **0** |
|  | **406** | **LNFPIII** | **0.000** | **0.003** | **0.0015** | **0.0001** |
|  | **406** | **LNH** | **0.000** | **0.000** | **0.0017** | **0.0001** |
|  | **406** | **LNTandLNnT** | **0.000** | **0.000** | **0.0017** | **0.0001** |
|  | 406 | LSTa | 0.005 | 0.147 | 0.0013 | 0.0001 |
|  | 406 | LSTb | 0.022 | 0.227 | 0.0012 | 0.0004 |
|  | 406 | LSTc | 0.038 | 0.279 | 0.0011 | 0.0004 |
|  | 416 | 2´FL | 0.029 | 0.194 | 0.0004 | 0.0001 |
|  | 416 | 3´SL | 0.048 | 0.441 | 0.0003 | 0.0001 |
|  | 416 | DFL | 0.011 | 0.108 | 0.0003 | 0 |
|  | **416** | **LNFPII** | **0.000** | **0.002** | **0.0006** | **0** |
|  | **416** | **LNFPIII** | **0.003** | **0.030** | **0.0003** | **0** |
|  | 416 | LNH | 0.006 | 0.072 | 0.0004 | 0.0000 |
|  | 416 | LNTandLNnT | 0.008 | 0.091 | 0.0004 | 0.0000 |
|  | 416 | LSTa | 0.026 | 0.294 | 0.0003 | 0 |
|  | **418** | **2´FL** | **0.000** | **0.004** | **0.0668** | **0.0211** |
|  | **418** | **DFL** | **0.000** | **0.000** | **0.0742** | **0.0022** |
|  | **418** | **LNDFHI** | **0.000** | **0.005** | **0.0704** | **0.0265** |
|  | 418 | LNFPI | 0.036 | 0.588 | 0.0737 | 0.0160 |
|  | **418** | **LNFPII** | **0.000** | **0.000** | **0.0967** | **0** |
|  | **418** | **LNFPIII** | **0.000** | **0.000** | **0.0757** | **0.0031** |
|  | **418** | **LNH** | **0.000** | **0.000** | **0.0884** | **0.0108** |
|  | **418** | **LNTandLNnT** | **0.000** | **0.000** | **0.0930** | **0.0105** |
|  | 418 | LSTa | 0.002 | 0.147 | 0.0654 | 0.0254 |
|  | 418 | LSTb | 0.001 | 0.070 | 0.0717 | 0.0217 |
|  | 418 | LSTc | 0.006 | 0.129 | 0.0699 | 0.0299 |
|  | **423** | **2´FL** | **0.001** | **0.022** | **0.0014** | **0.0001** |
|  | 423 | 3´SL | 0.027 | 0.410 | 0.0012 | 0.0005 |
|  | **423** | **DFL** | **0.000** | **0.002** | **0.0012** | **0** |
|  | 423 | LNDFHI | 0.007 | 0.110 | 0.0014 | 0.0004 |
|  | **423** | **LNFPII** | **0.000** | **0.000** | **0.0022** | **0** |
|  | **423** | **LNFPIII** | **0.000** | **0.005** | **0.0013** | **0.0001** |
|  | **423** | **LNH** | **0.000** | **0.000** | **0.0016** | **0.0001** |
|  | **423** | **LNTandLNnT** | **0.000** | **0.000** | **0.0016** | **0.0001** |
|  | 423 | LSTa | 0.008 | 0.169 | 0.0012 | 0.0001 |
|  | 423 | LSTb | 0.030 | 0.254 | 0.0011 | 0.0003 |
|  | 597 | 2´FL | 0.005 | 0.071 | 0.0013 | 0.0001 |
|  | 597 | 3´SL | 0.016 | 0.410 | 0.0012 | 0.0004 |
|  | **597** | **DFL** | **0.000** | **0.002** | **0.0012** | **0** |
|  | 597 | LNDFHI | 0.019 | 0.255 | 0.0013 | 0.0003 |
|  | **597** | **LNFPII** | **0.000** | **0.000** | **0.0021** | **0** |
|  | **597** | **LNFPIII** | **0.001** | **0.010** | **0.0013** | **0.0001** |
|  | **597** | **LNH** | **0.000** | **0.001** | **0.0015** | **0.0001** |
|  | **597** | **LNTandLNnT** | **0.000** | **0.002** | **0.0015** | **0.0001** |
|  | 597 | LSTa | 0.012 | 0.190 | 0.0011 | 0.0001 |
|  | 614 | 2´FL | 0.016 | 0.168 | 0.2053 | 0.1097 |
|  | **614** | **DFL** | **0.001** | **0.017** | **0.2532** | **0.1215** |
|  | 614 | LNDFHI | 0.039 | 0.377 | 0.2223 | 0.1482 |
|  | **614** | **LNFPII** | **0.000** | **0.002** | **0.2718** | **0.1180** |
|  | 614 | LNFPIII | 0.008 | 0.065 | 0.2290 | 0.1357 |
|  | **614** | **LNH** | **0.000** | **0.006** | **0.2418** | **0.1266** |
|  | **614** | **LNTandLNnT** | **0.002** | **0.024** | **0.2272** | **0.1167** |
|  | 630 | LSTa | 0.017 | 0.231 | 0.0001 | 0.0004 |
|  | **643** | **2´FL** | **0.001** | **0.018** | **0.0015** | **0.0002** |
|  | 643 | 3´SL | 0.035 | 0.410 | 0.0014 | 0.0005 |
|  | **643** | **DFL** | **0.000** | **0.001** | **0.0014** | **0** |
|  | 643 | LNDFHI | 0.002 | 0.059 | 0.0015 | 0.0004 |
|  | **643** | **LNFPII** | **0.000** | **0.000** | **0.0023** | **0** |
|  | **643** | **LNFPIII** | **0.000** | **0.003** | **0.0015** | **0.0001** |
|  | **643** | **LNH** | **0.000** | **0.000** | **0.0017** | **0.0001** |
|  | **643** | **LNTandLNnT** | **0.000** | **0.000** | **0.0017** | **0.0001** |
|  | 643 | LSTa | 0.005 | 0.147 | 0.0013 | 0.0001 |
|  | 643 | LSTb | 0.019 | 0.227 | 0.0012 | 0.0004 |
|  | 643 | LSTc | 0.036 | 0.279 | 0.0012 | 0.0005 |
| *g_Bilophila* | 280 | 2´FL | 0.047 | 0.270 | 0.0019 | 0.0002 |
|  | 280 | LNFPI | 0.024 | 0.588 | 0.0017 | 0 |
|  | 280 | LNFPV | 0.047 | 0.848 | 0.0005 | 0.0016 |
| *g_Blautia* | 509 | LNFPI | 0.024 | 0.588 | 0.0122 | 0 |
| *g_Citrobacter* | 297 | LNFPIII | 0.018 | 0.103 | 0.0000 | 0.0004 |
| *g_Clostridium* | 824 | 6´SL | 0.038 | 0.296 | 0.0022 | 0.0193 |
| *g_Escherichia-Shigella* | 316 | LNFPII | 0.029 | 0.210 | 0.0005 | 0.0002 |
|  | 316 | LNH | 0.044 | 0.327 | 0.0004 | 0.0002 |
| *g_Halomonas* | 373 | 3´SL | 0.017 | 0.410 | 0.0003 | 0.0012 |
|  | 373 | LNFPIII | 0.008 | 0.065 | 0.0009 | 0.0000 |
| *g_Lactobacillus* | **744** | **2´FL** | **0.000** | **0.004** | **0.0080** | **0.0013** |
|  | **744** | **DFL** | **0.000** | **0.001** | **0.0086** | **0.0002** |
|  | 744 | LNDFHI | 0.006 | 0.110 | 0.0057 | 0.0016 |
|  | 744 | LNTandLNnT | 0.035 | 0.255 | 0.0064 | 0.0048 |
|  | 744 | LSTb | 0.031 | 0.254 | 0.0036 | 0.0049 |
|  | 852 | LNFPII | 0.013 | 0.102 | 0.0207 | 0.0019 |
| *g_Lactococcus* | 697 | LNFPIII | 0.015 | 0.095 | 0.0004 | 0 |
|  | 698 | LNFPI | 0.007 | 0.588 | 0.0045 | 0.0010 |
|  | **698** | **LNFPIII** | **0.001** | **0.019** | **0.0044** | **0.0008** |
|  | 698 | LSTb | 0.047 | 0.273 | 0.0045 | 0.0020 |
| *g_Leuconostoc* | 962 | LNDFHI | 0.042 | 0.377 | 0.0005 | 0.0003 |
|  | 962 | LNFPIII | 0.011 | 0.074 | 0.0007 | 0.0001 |
|  | 962 | LSTb | 0.040 | 0.269 | 0.0008 | 0.0003 |
| *g_Odoribacter* | 241 | LNTandLNnT | 0.022 | 0.176 | 0.0018 | 0 |
| *g_Parabacteroides* | 181 | LNH | 0.012 | 0.125 | 0.0130 | 0.0437 |
|  | 196 | 2´FL | 0.040 | 0.251 | 0.0005 | 0 |
|  | 196 | 6´SL | 0.026 | 0.263 | 0.0004 | 0 |
|  | 196 | LSTb | 0.042 | 0.269 | 0.0003 | 0 |
|  | 196 | LSTc | 0.019 | 0.244 | 0.0004 | 0 |
|  | 249 | 2´FL | 0.029 | 0.194 | 0.0111 | 0.0002 |
| *g_Phascolarctobacterium* | 890 | 6´SL | 0.026 | 0.263 | 0.0036 | 0 |
|  | 890 | LSTb | 0.043 | 0.269 | 0.0052 | 0.0001 |
| *g_Ralstonia* | 553 | LNFPI | 0.041 | 0.588 | 0.0004 | 0 |
|  | 553 | LNFPIII | 0.034 | 0.162 | 0.0003 | 0 |
| *g_Rothia* | 430 | LNFPII | 0.036 | 0.226 | 0.0025 | 0.0003 |
| *g_Staphylococcus* | 591 | LSTb | 0.022 | 0.227 | 0 | 0.0003 |
|  | 591 | LSTc | 0.018 | 0.244 | 0 | 0.0002 |
|  | **721** | **6´SL** | **0.001** | **0.048** | **0.0087** | **0.0334** |
|  | 721 | LNTandLNnT | 0.047 | 0.295 | 0.0099 | 0.0181 |
|  | 721 | LSTa | 0.030 | 0.294 | 0.0155 | 0.0297 |
|  | 721 | LSTb | 0.002 | 0.080 | 0.0092 | 0.0239 |
|  | 721 | LSTc | 0.005 | 0.129 | 0.0078 | 0.0231 |
| *g_Streptococcus* | 667 | 2´FL | 0.021 | 0.168 | 0 | 0.0005 |
|  | 668 | DFL | 0.026 | 0.194 | 0.0034 | 0.0001 |
|  | **668** | **LNFPII** | **0.002** | **0.024** | **0.0036** | **0.0001** |
|  | 674 | DFL | 0.016 | 0.144 | 0.0019 | 0.0003 |
|  | 674 | LNFPIII | 0.042 | 0.181 | 0.0018 | 0.0006 |
|  | **685** | **6´SL** | **0.001** | **0.048** | **0.0138** | **0.0289** |
|  | 685 | LNFPII | 0.031 | 0.210 | 0.0194 | 0.0168 |
|  | 685 | LSTa | 0.033 | 0.294 | 0.0165 | 0.0360 |
|  | 685 | LSTc | 0.021 | 0.244 | 0.0150 | 0.0253 |
| *g_Veillonella* | 764 | 3´SL | 0.035 | 0.410 | 0.0018 | 0.0030 |
|  | 764 | LNFPII | 0.043 | 0.246 | 0.0038 | 0.0011 |
|  | 769 | 3´SL | 0.015 | 0.410 | 0.0011 | 0.0117 |
|  | 781 | 2´FL | 0.019 | 0.168 | 0.0018 | 0.0097 |
|  | 781 | 6´SL | 0.008 | 0.137 | 0.0022 | 0.0121 |
|  | 781 | LNTandLNnT | 0.010 | 0.100 | 0.0023 | 0.0118 |
| *g_Weissella* | 966 | LNFPIII | 0.034 | 0.162 | 0.0002 | 0 |
| *g_Aeribacillus* | 793 | LNFPIII | 0.010 | 0.074 | 0.0005 | 0 |
| f_Enterobacteriaceae_g_g | 302 | DFL | 0.024 | 0.192 | 0.0106 | 0.0782 |
| f_Enterobacteriaceae_g_g | 302 | LNFPII | 0.007 | 0.067 | 0.0014 | 0.0717 |
| f_Enterobacteriaceae_g_g | 302 | LNFPIII | 0.042 | 0.181 | 0.0182 | 0.0565 |
| f_Erysipelotrichaceae g_Incertae_Sedis | 714 | LNDFHI | 0.041 | 0.377 | 0.0001 | 0.0227 |
| f_Erysipelotrichaceae g_Incertae_Sedis | 714 | LNFPIII | 0.023 | 0.123 | 0.0121 | 0 |
| f_Erysipelotrichaceae *Incertae_Sedis* | 714 | LNH | 0.034 | 0.276 | 0.0001 | 0.0200 |
| f_Lachnospiraceae *Incertae_Sedis* | 496 | 6´SL | 0.040 | 0.296 | 0.0009 | 0 |
| f_Lachnospiraceae *Incertae_Sedis* | 496 | LSTc | 0.030 | 0.273 | 0.0010 | 0 |
| f_Streptococcaceae_g_g | **836** | **LNFPIII** | **0.002** | **0.019** | **0.0008** | **0.0000** |
| f_Streptococcaceae_g_g | 836 | LNFPII | 0.046 | 0.249 | 0.0009 | 0.0002 |

Table S7. DNA sequences of OTUs that differ in relative abundance between high and low consumer groups.

| **Taxonomy** | **OTU** | **Sequences (5’-3’)** |
| --- | --- | --- |
| *g_Aeribacillus* | 793 | TACGTAGGTGGCAAGCGTTGTCCGGAATTATTGGGCGTAAAGCGCGCGCAGGCGGTTCCTTAAGTCTGATGCCTGTTCGCTCCCCACGCTTTCGCGCCTCAGCGTCAGTTACAGGCCAGAGAGCCGCCTTCGCCACTGGTG |
| *g_Bacteroides* | 106 | TACGGAGGATCCGAGCGTTATCCGGATTTATTGGGTTTAAAGGGAGCGTAGATGGATGTTTAAGTCAGTTGCCTGTTTGATACCCACACTTTCGAGCCTCAATGTCAGTTGCAGCTTAGCAGGCTGCCTTCGCAATCGGAG |
|  | 133 | TACGGAGGATCCGAGCGTTATCCGGATTTATTGGGTTTAAAGGGAGCGTAGGCGGATTGTTAAGTCAGTTGCCTGTTTGATACCCACACTTTCGAGCATCAGCGTCAGTTACACTCCAGTGAGCTGCCTTCGCAATCGGAG |
|  | 142 | TACGGAGGATCCGAGCGTTATCCGGATTTATTGGGTTTAAAGGGAGCGTAGGCGGGTTGTTAAGTCAGTTGCCTGTTTGATACCCACACTTTCGAGCATCAGCGTCAGTTACAATCCAGTAAGCTGCCTTCGCAATCGGAG |
|  | 144 | TACGGAGGATCCGAGCGTTATCCGGATTTATTGGGTTTAAAGGGAGCGTAGGTGGACAGTTAAGTCAGTTGCCTGTTTGATACCCACACTTTCGAGCATCAGTGTCAGTTGCAGTCCAGTGAGCTGCCTTCGCAATCGGAG |
|  | 156 | TACGGAGGATCCGAGCGTTATCCGGATTTATTGGGTTTAAAGGGAGCGTAGGTGGATTGTTAAGTCAGTTGCCTGTTTGATACCCACACTTTCGAGCATCAGTGTCAGTAACAGTCTAGTGAGCTGCCTTCGCAATCGGAG |
|  | 227 | TACGGAGGATGCGAGCGTTATCCGGATTTATTGGGTTTAAAGGGAGCGTAGATGGATGTTTAAGTCAGTTGCCTGTTTGATACCCACACTTTCGAGCCTCAATGTCAGTTGCAGCTTAGCAGGCTGCCTTCGCAATCGGAG |
|  | 267 | TACGGAGGATTCGAGCGTTATCCGGATTTATTGGGTTTAAAGGGAGCGTAGATGGATGTTTAAGTCAGTTGCCTGTTTGATACCCACACTTTCGAGCCTCAATGTCAGTTGCAGCTTAGCAGGCTGCCTTCGCAATCGGAG |
| *g_Bifidobacterium* | 406 | TACGTAGGGCGCAAGCGTTATCCGGAATTATTGGGCGTAAAGGGCTCGTAGGCGGTTCGTCGCGTCCGGTGCCTGTTCGCTCCCCACGCTTTCGCTCCTCAGCGTCAGTAACGGCCCAGAGACCTGCCTTCGCCATTGGTG |
|  | 416 | TACGTAGGGCGCAAGCGTTATCCGGATTTATTGGGCGTAAAGGGCTCGTAGGCGGCTCGTCGCGTCCGGTGCCTGTTCGCTCCCCACGCTTTCGCTCCTCAGCGTCAGTAACGGCCCAGAGACCTGCCTTCGCCATCGGTG |
|  | 418 | TACGTAGGGCGCAAGCGTTATCCGGATTTATTGGGCGTAAAGGGCTCGTAGGCGGCTCGTCGCGTCCGGTGCCTGTTCGCTCCCCACGCTTTCGCTCCTCAGCGTCAGTGACGGCCCAGAGACCTGCCTTCGCCATCGGTG |
|  | 423 | TACGTAGGGCGCAAGCGTTATCCGGATTTATTGGGCGTAAAGGGCTCGTAGGCGGTTCGTCGCGTCCGGTGCCTGTTCGCTCCCCACGCTTTCGCTCCTCAGCGTCAGTAACGGCCCAGAGACCTGCCTTCGCCATTGGTG |
|  | 597 | TACGTAGGGTGCAAGCGTTATCCGGAATTATTGGGCGTAAAGGGCTCGTAGGCGGCTCGTCGCGTCCGGTGCCTGTTCGCTCCCCACGCTTTCGCTCCTCAGCGTCAGTGACGGCCCAGAGACCTGCCTTCGCCATCGGTG |
|  | 614 | TACGTAGGGTGCAAGCGTTATCCGGAATTATTGGGCGTAAAGGGCTCGTAGGCGGTTCGTCGCGTCCGGTGCCTGTTCGCTCCCCACGCTTTCGCTCCTCAGCGTCAGTAACGGCCCAGAGACCTGCCTTCGCCATTGGTG |
|  | 630 | TACGTAGGGTGCAAGCGTTATCCGGAATTATTGGGCGTAAAGGGCTCGTAGGCGGTTCGTCGCGTCCGGTGCCTGTTTGCTCCCCACGCTTTCGCTCCTCAGCGTCAGTAACGGCCCAGAGACCTGCCTTCGCCATTGGTG |
|  | 643 | TACGTAGGGTGCAAGCGTTATCCGGATTTATTGGGCGTAAAGGGCTCGTAGGCGGCTCGTCGCGTCCGGTGCCTGTTCGCTCCCCACGCTTTCGCTCCTCAGCGTCAGTGACGGCCCAGAGACCTGCCTTCGCCATCGGTG |
| *g_Bilophila* | 280 | TACGGAGGGTGCAAGCGTTAATCGGAATCACTGGGCGTAAAGCGCACGTAGGCGGCTTGGTAAGTCAGGGGCCTGTTTGCTACCCACGCTTTCGCACCTCAGCGTCAGTTACCGTCCAGGTGGCCGCCTTCGCCACCGGTG |
| *g_Blautia* | 509 | TACGTAGGGGGCAAGCGTTATCCGGATTTACTGGGTGTAAAGGGAGCGTAGACGGTGTGGCAAGTCTGATGCCTGTTTGCTCCCCACGCTTTCGAGCCTCAACGTCAGTTACCGTCCAGTAAGCCGCCTTCGCCACTGGTG |
| *g_Citrobacter* | 297 | TACGGAGGGTGCAAGCGTTAATCGGAATTACTGGGCGTAAAGCGCACGCAGGCGGTCTGTCAAGTCGGATGCCTGTTTGCTCCCCACGCTTTCGCACCTGAGCGTCAGTCTTCGTCCAGGGGGCCGCCTTCGCCACCGGTA |
| *g_Clostridium* | 824 | TACGTAGGTGGCAAGCGTTGTCCGGATTTACTGGGCGTAAAGGGAGCGTAGGCGGATTTTTAAGTGGGATGCCTGTTTGCTCCCCACGCTTTCGAGCCTCAGCGTCAGTTACAGTCCAGAAAGTCGCCTTCGCCACTGGTG |
| *g_Escherichia-Shigella* | 316 | TACGGAGGGTGCAAGCGTTAATCGGAATTACTGGGCGTAAAGCGCACGCAGGCGGTTTGTTAAGTCAGATGCCTGTTCGCTCCCCACGCTTTCGCACCTGAGCGTCAGTCTTCGTCCAGGGGGCCGCCTTCGCCACCGGTA |
| *g_Halomonas* | 373 | TACGGAGGGTGCGAGCGTTAATCGGAATTACTGGGCGTAAAGCGCGCGTAGGCGGTCTGATAAGCCGGTTGCCTGTTTGCTACCCACGCTTTCGCACCTCAGCGTCAGTGTCAGTCCAGAAGGCCGCCTTCGCCACTGGTA |
| *g_Lactobacillus* | 744 | TACGTAGGTGGCAAGCGTTATCCGGATTTATTGGGCGTAAAGCGAGCGCAGGCGGTTTTTTAAGTCTGATGCCTGTTCGCTACCCATGCTTTCGAGCCTCAGCGTCAGTTACAGACCAGACAGCCGCCTTCGCCACTGGTG |
|  | 852 | TACGTAGGTGGCAAGCGTTGTCCGGATTTATTGGGCGTAAAGCGAGTGCAGGCGGTTCAATAAGTCTGATGCCTGTTCGCTACCCATGCTTTCGAGCCTCAGCGTCAGTTGCAGACCAGAGAGCCGCCTTCGCCACTGGTG |
| *g_Lactococcus* | 697 | TACGTAGGTCCCGAGCGTTGTCCGGATTTATTGGGCGTAAAGCGAGCGCAGGTGGTTTAATAAGTCTGATGCCTGTTTGCTACCCACGCTTTCGAGCCTCAGTGTCAGTTACAGTCCAGAGAGCCGCTTTCGCCACCGGTG |
|  | 698 | TACGTAGGTCCCGAGCGTTGTCCGGATTTATTGGGCGTAAAGCGAGCGCAGGTGGTTTATTAAGTCTGGTGCCTGTTTGCTCCCCACGCTTTCGAGCCTCAGTGTCAGTTACAGGCCAGAGAGCCGCTTTCGCCACCGGTG |
| *g_Leuconostoc* | 962 | TACGTATGTCCCGAGCGTTATCCGGATTTATTGGGCGTAAAGCGAGCGCAGACGGTTGATTAAGTCTGATGCCTGTTTGCTACCCACACTTTCGAGCCTCAACGTCAGTTGTTGTCCAGTAAGCCGCCTTCGCCACTGGTG |
| *g_Odoribacter* | 241 | TACGGAGGATGCGAGCGTTATCCGGATTTATTGGGTTTAAAGGGTGCGTAGGCGGTTTATTAAGTTAGTGGCCTGTTCGCTACCCACGCTCTCGTGCATCAGCGTCAGTTACAGTCTGGTAAGCTGCCTTCGCTATCGGAG |
| *g_Parabacteroides* | 181 | TACGGAGGATCCGAGCGTTATCCGGATTTATTGGGTTTAAAGGGTGCGTAGGCGGCCTTTTAAGTCAGCGGCCTGTTTGATCCCCACGCTTTCGTGCATCAGCGTCAGTCATGGCTTGGCAGGCTGCCTTCGCAATCGGGG |
|  | 196 | TACGGAGGATCCGAGCGTTATCCGGATTTATTGGGTTTAAAGGGTGCGTAGGTGGTGATTTAAGTCAGCGGCCTGTTTGATCCCCACGCTTTCGTGCTTCAGTGTCAGTTATGGTTTAGTAAGCTGCCTTCGCAATCGGAG |
|  | 249 | TACGGAGGATGCGAGCGTTATCCGGATTTATTGGGTTTAAAGGGTGCGTAGGTGGTGATTTAAGTCAGCGGCCTGTTTGATCCCCACGCTTTCGTGCTTCAGTGTCAGTTATGGTTTAGTAAGCTGCCTTCGCAATCGGAG |
| *g_Phascolarctobacterium* | 890 | TACGTAGGTGGCGAGCGTTGTCCGGAATTATTGGGCGTAAAGAGCATGTAGGCGGCTTAATAAGTCGAGCGCCCGTTCGCTACCCTGGCTTTCGCATCTCAGCGTCAGACACAGTCCAGAAAGGCGCCTTCGCCACTGGTG |
| *g_Ralstonia* | 553 | TACGTAGGGTCCAAGCGTTAATCGGAATTACTGGGCGTAAAGCGTGCGCAGGCGGTTGTGCAAGACCGATGCCTGTTTGCTCCCCACGCTTTCGTGCATGAGCGTCAGTGTTATCCCAGGGGGCTGCCTTCGCCATCGGTA |
| *g_Rothia* | 430 | TACGTAGGGCGCGAGCGTTGTCCGGAATTATTGGGCGTAAAGAGCTTGTAGGCGGTTTGTCGCGTCTGCTGCCTGTTCGCTCCCCATGCTTTCGCTTCTCAGCGTCAGTTACAGCCCAGAGACCTGCCTTCGCCATCGGTG |
| *g_Staphylococcus* | 591 | TACGTAGGGTGCAAGCGTTATCCGGAATTATTGGGCGTAAAGCGCGCGTAGGCGGTTTTTTAAGTCTGATGCCTGTTTGATCCCCACGCTTTCGCACATCAGCGTCAGTTACAGACCAGAAAGTCGCCTTCGCCACTGGTG |
|  | 721 | TACGTAGGTGGCAAGCGTTATCCGGAATTATTGGGCGTAAAGCGCGCGTAGGCGGTTTTTTAAGTCTGATGCCTGTTTGATCCCCACGCTTTCGCACATCAGCGTCAGTTACAGACCAGAAAGTCGCCTTCGCCACTGGTG |
| *g_Streptococcus* | 667 | TACGTAGGTCCCGAGCGTTATCCGGATTTATTGGGCGTAAAGCGAGCGCAGGCGGTTAGATAAGTCTGAAGCCTGTTTGCTCCCCACGCTTTCGAGCCTCAGCGTCAGTTACAAGCCAGAGAGCCGCTTTCGCCACCGGTG |
|  | 668 | TACGTAGGTCCCGAGCGTTATCCGGATTTATTGGGCGTAAAGCGAGCGCAGGCGGTTAGATAAGTCTGAAGCCTGTTTGCTCCCCACGCTTTCGAGCCTCAGCGTCAGTTACAGACCAGAGAGCCGCTTTCGCCACCGGTG |
|  | 674 | TACGTAGGTCCCGAGCGTTGTCCGGATTTATTGGGCGTAAAGCGAGCGCAGGCGGTTAGATAAGTCTGAAGCCTGTTTGCTCCCCACGCTTTCGAGCCTCAGCGTCAGTTACAAGCCAGAGAGCCGCTTTCGCCACCGGTG |
|  | 685 | TACGTAGGTCCCGAGCGTTGTCCGGATTTATTGGGCGTAAAGCGAGCGCAGGCGGTTTGATAAGTCTGAAGCCTGTTCGCTCCCCACGCTTTCGAGCCTCAGCGTCAGTTACAGACCAGAGAGCCGCTTTCGCCACCGGTG |
| *g_Veillonella* | 764 | TACGTAGGTGGCAAGCGTTGTCCGGAATTATTGGGCGTAAAGCGCGCGCAGGCGGATAGGTCAGTCTGTCTCCCGTTCGCTCCCCTGGCTTTCGCGCCTCAGCGTCAGTTTTCGTCCAGAAAGTCGCCTTCGCCACTGGTG |
|  | 769 | TACGTAGGTGGCAAGCGTTGTCCGGAATTATTGGGCGTAAAGCGCGCGCAGGCGGATCAGTCAGTCTGTCTCCCGTTCGCTCCCCTGGCTTTCGCGCCTCAGCGTCAGTTTTCGTCCAGAAAGTCGCCTTCGCCACTGGTG |
|  | 781 | TACGTAGGTGGCAAGCGTTGTCCGGAATTATTGGGCGTAAAGCGCGCGCAGGCGGATTGGTCAGTCTGTCTCCCGTTCGCTCCCCTGGCTTTCGCGCCTCAGCGTCAGTTTTCGTCCAGAAAGTCGCCTTCGCCACTGGTG |
| *g_Weissella* | 966 | TACGTATGTTCCAAGCGTTATCCGGATTTATTGGGCGTAAAGCGAGCGCAGACGGTTATTTAAGTCTGAAGCCTGTTTGCTACCCACACTTTCGAGCCTCAACGTCAGTTACAGTCCAGAAAGCCGCCTTCGCCACTGGTG |
| f_Enterobacteriaceae | 302 | TACGGAGGGTGCAAGCGTTAATCGGAATTACTGGGCGTAAAGCGCACGCAGGCGGTCTGTCAAGTCGGATGCCTGTTTGCTCCCCACGCTTTCGCACCTGAGCGTCAGTCTTTGTCCAGGGGGCCGCCTTCGCCACCGGTA |
| f_Erysipelotrichaceae *Incertae_Sedis* | 714 | TACGTAGGTGGCAAGCGTTATCCGGAATTATTGGGCGTAAAGAGGGAGCAGGCGGCAGCAAGGGTCTGTGGCCTATTTGCTCCCCACGCTTTCGGGACTGAGCGTCAGTTGCAGGCCAGATCGTCGCCTTCGCCACTGGTG |
| f_Lachnospiraceae *Incertae_Sedis* | 496 | TACGTAGGGGGCAAGCGTTATCCGGATTTACTGGGTGTAAAGGGAGCGTAGACGGCGAAGCAAGTCTGAAGCCTGTTTGCTCCCCACGCTTTCGAGCCTCAACGTCAGTTATCGTCCAGTAAGCCGCCTTCGCCACTGGTG |
| f_Streptococcaceae | 836 | TACGTAGGTGGCAAGCGTTGTCCGGATTTATTGGGCGTAAAGCGAGCGCAGGCGGTTCATTAAGTCTGATGCCTGTTTGCTCCCCACGCTTTCGAGCCTCAGCGTCAGTTACAGTCCAGAGAGCCGCTTTCGCCTCCGGTG |
